# Supplementary material for: An accurate and efficient measure of welfare tradeoff ratios
Source: PLoS One. 2025 May 27;20(5):e0322410. doi: 10.1371/journal.pone.0322410 (PMC12112420; doi:10.1371/journal.pone.0322410)
Supplement: S1 Appendix — (PDF) [file pone.0322410.s001.pdf]

S1 Appendix
Formal derivation of the Lambda Slider

1 The general form

Suppose we have a curve on the w_s–w_t space defined by $w_s = f(w_t)$, $w_t \in [w_{min}, w_{max}]$, and f is everywhere differentiable on $w_t \in (w_{min}, w_{max})$ and strictly concave (or, equivalently, f' is strictly decreasing), from which we can deduce that f' is continuous and invertible. We can rewrite the utility (Eq (1)) as a function of w_t:

$$\begin{aligned} u(w_t) &= w_s + \lambda w_t \\ &= f(w_t) + \lambda w_t\,, \quad w_t \in [w_{min}, w_{max}]\,. \end{aligned}$$

Then we have

$$u'(w_t) = f'(w_t) + \lambda\,, \quad w_t \in (w_{min}, w_{max})\,.$$

Since $f'(w_t)$ is continuous and strictly decreasing, we have

$$u'(w_t) \begin{cases} > 0 & w_t \in \left(w_{min}, f'^{-1}(-\lambda)\right) \\ = 0 & w_t = f'^{-1}(-\lambda) \\ < 0 & w_t \in \left(f'^{-1}(-\lambda), w_{max}\right) \end{cases}\,,$$

as long as $w_{min} < f'^{-1}(-\lambda) < w_{max}$, or, equivalently, $-f'(w_{min}) < \lambda < -f'(w_{max})$. Therefore,

$$w_t^* = \underset{w_t \in [w_{min}, w_{max}]}{\arg\max}\ u(w_t) = f'^{-1}(-\lambda)\,, \quad \forall \lambda \in \left(-f'(w_{min}), -f'(w_{max})\right).$$

In other words, there is a one-to-one correspondence between $\lambda \in \left(-f'(w_{min}), -f'(w_{max})\right)$ and points on the curve that a utility-maximizing participant will choose.

To derive a slider and two payoff functions from this curve, we can parameterize the curve as

$$\begin{aligned} w_t &= g(x)\,, \\ w_s &= f\left(g(x)\right), \\ x &\in [x_{min}, x_{max}]\,, \end{aligned}$$

where x is the slider position, x_{min} and x_{max} are the boundaries of the slider, and g is a con-

tinuous and strictly monotonic (and thus invertible) function. The slider (with the two payoff functions) derived in such a way is called a Lambda Slider. If g is strictly increasing, we have $x_{\mathrm{min}} = g^{-1}(w_{\mathrm{min}})$ and $x_{\mathrm{max}} = g^{-1}(w_{\mathrm{max}})$, and the relationship is reversed if g is strictly decreasing. Then the slider position that the participant (with $\lambda \in \big(-f'(w_{\mathrm{min}}), -f'(w_{\mathrm{max}})\big)$) will choose is

$$\begin{aligned} x^* &= g^{-1}(w_{\mathrm{t}}^*) \\ &= g^{-1}\big(f'^{-1}(-\lambda)\big). \end{aligned}$$

Let $h(\lambda) = g^{-1}\big(f'^{-1}(-\lambda)\big)$. Since both g^{-1} and f'^{-1} are continuous and strictly monotonic functions, h is also a continuous and strictly monotonic function, so there is a one-to-one correspondence between x^* and λ.

2 Quadratic Lambda Slider

If we select g such that $g(x) = f'^{-1}(-x)$, we have

$$\begin{aligned} x^* &= -f'\big(f'^{-1}(-\lambda)\big) \\ &= -(-\lambda) \\ &= \lambda, \end{aligned}$$

in which case h is the identity function.

What are the simplest f and g such that h is the identity function? Can f, g, or $f \circ g$ (the payoff function for "self") be linear? Since f is strictly concave, it cannot be linear. In order to measure both positive and negative λs, $f'(w_{\mathrm{min}})$ and $f'(w_{\mathrm{max}})$ need to have different signs, which means f cannot be monotonic. Since g is monotonic, $f \circ g$ cannot be monotonic, and thus cannot be linear. Therefore, only g can be linear.

Let $w_{\mathrm{t}} = g(x) = Ax + B$, $A > 0$. Given $g(x) = f'^{-1}(-x)$, we have

$$\begin{aligned} & Ax + B = f'^{-1}(-x) \\ \Rightarrow\quad & f'(Ax + B) = -x \\ \Rightarrow\quad & f'(w_{\mathrm{t}}) = -\frac{w_{\mathrm{t}} - B}{A} \\ \Rightarrow\quad & f(w_{\mathrm{t}}) = \int -\frac{w_{\mathrm{t}} - B}{A}\, \mathrm{d}w_{\mathrm{t}} \\ & \qquad = -\frac{1}{2A}(w_{\mathrm{t}} - B)^2 + C, \end{aligned}$$

where C is an arbitrary constant, and

$$\begin{aligned} w_{\mathrm{s}} &= f\big(g(x)\big) \\ &= -\frac{1}{2A}\big((Ax+B)-B\big)^2 + C \\ &= -\frac{A}{2}x^2 + C\,. \end{aligned}$$

Letting $A = 2a$, $B = b_{\mathrm{t}}$ and $C = b_{\mathrm{s}}$, we get the same payoff functions as Eqs (2) and (3).
