# Supplementary material for: An accurate and efficient measure of welfare tradeoff ratios
Source: PLoS One. 2025 May 27;20(5):e0322410. doi: 10.1371/journal.pone.0322410 (PMC12112420; doi:10.1371/journal.pone.0322410)
Supplement: S2 Appendix — (PDF) [file pone.0322410.s002.pdf]

S2 Appendix
Circle Test and circular Lambda Slider

Sonnemans et al. [1] introduced a "Circle Test" to measure participants' social value orientations (or λs). Participants are presented with a w_s–w_t plane and are asked to choose a point on a circle defined by $w_s^2 + w_t^2 = 1000$. The Circle Test has the same underlying logic as the Lambda Slider, and, theoretically, there is also a one-to-one correspondence between the participant's potential λs and points on the (right half of the) circle.

To see this, we can create a Lambda Slider that is almost the same as the Circle Test, by selecting f that defines a half circle on the w_s–w_t plane and verifying that f satisfies the requirements of a Lambda Slider. Instead of writing out f directly, it is easier to parameterize the curve with θ:

$$\begin{aligned} w_s &= a\cos\theta + b_s, \\ w_t &= a\sin\theta + b_t, \\ \theta &\in \left[-\frac{\pi}{2}, \frac{\pi}{2}\right], \end{aligned}$$

where $a > 0$, b_s and b_t are arbitrary scale and shift parameters. Then we have

$$\begin{aligned} f'(w_t) &= \frac{\mathrm{d}w_s}{\mathrm{d}w_t} \\ &= \frac{\mathrm{d}w_s}{\mathrm{d}\theta} \bigg/ \frac{\mathrm{d}w_t}{\mathrm{d}\theta} \\ &= \frac{-\sin\theta}{\cos\theta} \\ &= -\tan\theta, \end{aligned}$$

which confirms that f is everywhere differentiable on $(-a + b_t, a + b_t)$ and strictly concave. Since the relationship between w_t and θ is bijective, we can define a unique θ^* according to

$$w_t^* = a\sin\theta^* + b_t$$

and have

$$\begin{aligned} w_t^* &= f'^{-1}(-\lambda) \\ \Rightarrow \quad f'(w_t^*) &= -\lambda \\ \Rightarrow \quad -\tan\theta^* &= -\lambda \\ \Rightarrow \quad \theta^* &= \arctan\lambda\,. \end{aligned}$$

Based on this curve, we can define a Lambda Slider by letting $x = \theta$:

$$w_s(x) = a\cos x + b_s\,, \tag{13}$$

$$w_t(x) = a\sin x + b_t\,, \tag{14}$$

$$x \in \left[-\frac{\pi}{2}, \frac{\pi}{2}\right].$$

We call such a Lambda Slider the “circular Lambda Slider”. Applying the utility definition of Eq (1), it can be easily verified that

$$x^* = \arg\max_{x\in[-\frac{\pi}{2},\frac{\pi}{2}]} u(x) = \arctan\lambda\,, \quad \forall\lambda \in \mathbb{R}\,. \tag{15}$$

We see that the circular Lambda Slider can measure an infinite range of λ, while the quadratic Lambda Slider cannot (due to the constraint of an identity function between λ and x).

Although the Circle Test has the same underlying logic as the Lambda Slider, there are three limitations in [1]’s presentation of the Circle Test. First, they developed the Circle Test as an intuitive extension of the Ring Measure [2] without linking the measured angle to λ itself. Second, [1] only used the Circle Test as a tool without testing its psychometric properties. Third, the Circle Test involves negative payoffs because the payoff structure is defined by $w_s^2 + w_t^2 = 1000$. This seems to result from a direct influence of the Ring Measure [2]. It is well known that people interpret gains and losses differently [3] and mixing positive and negative payoffs might exacerbate the nonlinearity in the relationship between perceived welfare and payoffs, biasing the measurements. In our Lambda Slider, we can select the shift parameters b_s and b_t such that the payoffs are always positive or always negative, and we only used positive payoffs in our experiments.

An important difference between the Circle Test and the circular Lambda Slider is that the payoff structure of the Circle Test is a full circle, while the payoff structure of the circular Lambda Slider is a half circle. This raises two related questions: (a) How can we explain choices (if any) made on the left half of the circle in the Circle Test? (b) What happens when

we extend the range of the circular Lambda Slider to $x > \frac{\pi}{2}$ and/or $x < -\frac{\pi}{2}$ while keeping the functional forms of Eqs (13) and (14)[1]? If we restrict the utility function to be a linear combination of w_s and w_t, the participant must have a zero or negative coefficient on w_s in her utility function in order to choose $x \in \left(-\pi, -\frac{\pi}{2}\right] \cup \left[\frac{\pi}{2}, \pi\right]$. In reality and in experimental data (e.g., [1]), it is unlikely for someone to have a zero or negative coefficient on w_s (i.e., all else being equal, the person is indifferent about her own payoff or prefers a lower payoff for herself). Hence in this paper we mostly restrict ourselves to the utility function in the form of Eq (1), which entails that any extension of the circular Lambda Slider beyond a half circle is useless because those points do not correspond to any λ.

However, it has been shown that people can perceive and make predictions based on social value orientations of altruism ($x = \frac{\pi}{2}$), martyrdom ($x = \frac{3\pi}{4}$), masochism ($x = \pi$), sadomasochism ($x = -\frac{3\pi}{4}$) and aggression ($x = -\frac{\pi}{2}$), which involve zero or negative coefficients on w_s, although their ability to understand these motivations is generally worse than motivations with positive coefficients on w_s [4]. We can capture such "abnormal" motivations with a different parameterization of the utility function, such as

$$u = w_s \cos\phi + w_t \sin\phi, \tag{16}$$

where $\phi \in (-\pi, \pi]$ is a parameter analogous to λ, and this utility function is equivalent to Eq (1) (up to a scaling factor) given $\lambda = \tan\phi$ for $\phi \in \left(-\frac{\pi}{2}, \frac{\pi}{2}\right)$. ϕ is also equivalent to θ_M in [5]. Then we can define the "Phi Slider", which is a (potentially) accurate and efficient measure of ϕ, and the "circular Phi Slider", which has the same payoff functions as Eqs. (13) and (14) but a wider range over x. For the circular Phi Slider, we have

$$x^* = \underset{x \in (-\pi, \pi]}{\arg\max}\, u(x) = \phi, \quad \forall \phi \in (-\pi, \pi],$$

so there is a one-to-one correspondence (an identity function) between the slider position a participant chooses and her ϕ.

On the other hand, extending the range of the circular Lambda Slider might be useful even if we are committed to the utility function of Eq (1). Participants are often drawn to the boundaries of a finite-length slider, even when those points do not strictly maximize their utility. If the circular Lambda Slider has a range of $x \in \left[-\frac{\pi}{2}, \frac{\pi}{2}\right]$, participants' choices near the boundaries of the slider are likely to be biased toward the boundaries, and many responses would correspond to $\lambda = \pm\infty$. To eliminate the salient points of $x = \pm\frac{\pi}{2}$ on the

[1]In this case the slider is no longer a Lambda Slider as defined in S1 Appendix, because the curve on the w_s–w_t plane cannot be written in the form of $w_s = f(w_t)$. We need a more general definition of a "ϕ slider" that can measure ϕ as defined in Eq (16), which we do not elaborate in the current paper.

slider, the experimenter can extend the range of the slider such that the boundaries (e.g., $x = \pm\frac{2\pi}{3}$) are sufficiently discouraged for typical social motivations (i.e., positive coefficient on w_s) and responses near $x = \pm\frac{\pi}{2}$ are minimally biased.

Another difference between the Circle Test and the circular Lambda Slider is that the Circle Test is presented as a circle on the computer screen, and participants are asked to choose a point on the circle, while the Lambda Slider is presented as a linear slider. In general, a Lambda Slider (or a Phi Slider) can be presented either as a linear slider with two bars of varying lengths indicating the payoffs (1D presentation), or as a curve on the w_s–w_t plane (2D presentation). We think that neither of these two presentations is intrinsically better, but for the Phi Slider, the 2D presentation seems more intuitive when the range of ϕ we want to measure is $(-\pi, \pi]$, while the 1D presentation seems more intuitive when the range of ϕ we want to measure is smaller, such as $\left(-\frac{\pi}{2}, \frac{\pi}{2}\right)$ (in which case we can measure λ instead of ϕ). It is possible that one of these two presentations has better psychometric properties than the other, which future research can investigate.

References

1. Sonnemans J, van Dijk F, van Winden F. On the dynamics of social ties structures in groups. Journal of Economic Psychology. 2006;27(2):187–204.

2. Liebrand WBG. The effect of social motives, communication and group size on behaviour in an N-person multi-stage mixed-motive game. European Journal of Social Psychology. 1984;14(3):239–264.

3. Kahneman D, Tversky A. Prospect theory: An analysis of decision under risk. Econometrica. 1979;47(2):263–292.

4. Maki JE, Thorngate WB, McClintock CG. Prediction and perception of social motives. Journal of Personality and Social Psychology. 1979;37(2):203–220.

5. Griesinger DW, Livingston Jr JW. Toward a model of interpersonal motivation in experimental games. Behavioral Science. 1973;18(3):173–188.
