# Supplementary material for: An accurate and efficient measure of welfare tradeoff ratios
Source: PLoS One. 2025 May 27;20(5):e0322410. doi: 10.1371/journal.pone.0322410 (PMC12112420; doi:10.1371/journal.pone.0322410)
Supplement: S3 Appendix — (PDF) [file pone.0322410.s003.pdf]

S3 Appendix
Payoff functions in Experiments 2 and 3

In Experiment 2, we would like the payoff functions of the base, positive-shift (pos), and negative-shift (neg) sliders (in the form of Eqs (2)–(4)) to have the following properties:

1. The range of x on the base slider is $[-2,2]$ (same as Experiment 1).
2. The range of x on the pos (neg) slider is a constant shift $d > 0$ upward (downward) from the base slider, and the larger d is, the better.
3. The range of w_{t} is $[5,95]$ on any slider.
4. The range of w_{s} is narrower than $[5,95]$ on any slider.
5. Let χ^*_{base}, χ^*_{pos} and χ^*_{neg} be the raw slider positions corresponding to $w_{\mathrm{s}} = w_{\mathrm{t}}$ on the three sliders, respectively (the equal-payoff points). H_λ predicts $\chi_{\mathrm{pos}} = \chi_{\mathrm{base}} - d$ and $\chi_{\mathrm{neg}} = \chi_{\mathrm{base}} + d$ while H_χ predicts $\chi_{\mathrm{pos}} = \chi_{\mathrm{neg}} = \chi_{\mathrm{base}}$. We would like χ^*_{base}, χ^*_{pos} and χ^*_{neg} to be halfway between the predictions of H_λ and H_χ so that inequity-averse responses do not bias toward one of the hypotheses, and thus $\chi^*_{\mathrm{pos}} = \chi^*_{\mathrm{base}} - \frac{d}{2}$ and $\chi^*_{\mathrm{neg}} = \chi^*_{\mathrm{base}} + \frac{d}{2}$.

We find that 0.75 is almost the maximum value d can have to satisfy all these constraints, so we set $d = 0.75$ and use the following parameters:

$$\begin{aligned}
\text{base:}\quad & a = 11.25, & b_{\mathrm{s}} &= 90, & b_{\mathrm{t}} &= 50,\\
\text{pos:}\quad & a = 11.25, & b_{\mathrm{s}} &= 92.716, & b_{\mathrm{t}} &= 33.125,\\
\text{neg:}\quad & a = 11.25, & b_{\mathrm{s}} &= 90.448, & b_{\mathrm{t}} &= 66.875.
\end{aligned}$$

Fig 6 confirms that these sliders satisfy constraints 1–4. In Fig 7, a tight cluster of points halfway between the predictions of the two hypotheses corresponds to the inequity-averse responses, which confirms that the sliders satisfy constraint 5.

In Experiment 3, all three sliders have the same range $[-2,2]$ and scale $a = 7$, but different offsets:

$$\begin{aligned}
\text{balanced:}\quad & b_{\mathrm{s}} = 64, & b_{\mathrm{t}} &= 50,\\
\text{self-more:}\quad & b_{\mathrm{s}} = 95, & b_{\mathrm{t}} &= 33,\\
\text{target-more:}\quad & b_{\mathrm{s}} = 33, & b_{\mathrm{t}} &= 67.
\end{aligned}$$
