# Supplementary material for: An accurate and efficient measure of welfare tradeoff ratios
Source: PLoS One. 2025 May 27;20(5):e0322410. doi: 10.1371/journal.pone.0322410 (PMC12112420; doi:10.1371/journal.pone.0322410)
Supplement: S4 Appendix — (PDF) [file pone.0322410.s004.pdf]

S4 Appendix
Model specifications

1 Experiment 1

1.1 Test–retest reliability

The two measurements for each participant–target combination i form a data vector $\boldsymbol{x}_i$. The data vectors are assumed to be sampled i.i.d. from a bivariate normal distribution where the two variables have the same mean and standard deviation:[1]

$$\begin{aligned}\boldsymbol{x}_i &\sim N(\boldsymbol{\mu}, \boldsymbol{\Sigma}),\\ \boldsymbol{x}_i &= (x_{i1}, x_{i2})^\top,\\ \boldsymbol{\mu} &= (\mu, \mu)^\top,\\ \boldsymbol{\Sigma} &= \sigma^2 \begin{pmatrix} 1 & \rho \\ \rho & 1 \end{pmatrix},\end{aligned}$$

where ρ is the correlation parameter representing the test–retest reliability. We set the priors to be

$$\begin{aligned}\mu &\sim N(0, 2),\\ \sigma &\sim \text{Lognormal}\big(\ln(1), 1\big),\\ \rho &\sim \text{Uniform}(-1, 1)\end{aligned}$$

for the Lambda Slider data, and

$$\begin{aligned}\mu &\sim N(20, 40),\\ \sigma &\sim \text{Lognormal}\big(\ln(20), 1\big),\\ \rho &\sim \text{Uniform}(-1, 1)\end{aligned}$$

for the SVO Slider Measure data. Elements of $\boldsymbol{x}_i$ are restricted to a range ($[-2, 2]$ for λ and $[-16.26°, 61.39°]$ for SVO°), so we treat data points that lie on the boundaries as censored data. For example, if $\lambda_{i1} = 2$ and $\lambda_{i2} = 1.8$, λ_{i2} will be used as data in the model, but λ_{i1} will

[1]In this paper, univariate normal distributions, denoted by $N(\mu, \sigma)$, are parameterized by their standard deviations instead of variances; multivariate normal distributions, denoted by $N(\boldsymbol{\mu}, \boldsymbol{\Sigma})$, are parameterized by their covariance matrices as usual. Log-normal distributions, denoted by Lognormal(μ, σ), are parameterized by their means and standard deviations in the log space.

be a parameter with a lower bound of 2, which will be included in the posterior samples along with the other model parameters. We fit the model using RStan [1,2] with the default sampling parameters.

1.2 Lambda Slider vs. SVO Slider Measure

For each participant–target combination i, the two measurements on the Lambda Slider (denoted by λ) and the two measurement on the SVO Slider Measure (denoted by ν) form a data vector $\boldsymbol{x}_i$. The data vectors are assumed to be sampled i.i.d. from a 4-variate normal distribution with certain constraints:

$$
\begin{aligned}
\boldsymbol{x}_i &\sim N(\boldsymbol{\mu}, \boldsymbol{\Sigma}), \\
\boldsymbol{x}_i &= (\lambda_{i1}, \lambda_{i2}, \nu_{i1}, \nu_{i2})^\top, \\
\boldsymbol{\mu} &= (\mu_\lambda, \mu_\lambda, \mu_\nu, \mu_\nu)^\top, \\
\boldsymbol{\Sigma} &= \begin{pmatrix} \sigma_\lambda^2 & \sigma_{\lambda\lambda} & \sigma_{\lambda\nu} & \sigma_{\lambda\nu} \\ \sigma_{\lambda\lambda} & \sigma_\lambda^2 & \sigma_{\lambda\nu} & \sigma_{\lambda\nu} \\ \sigma_{\lambda\nu} & \sigma_{\lambda\nu} & \sigma_\nu^2 & \sigma_{\nu\nu} \\ \sigma_{\lambda\nu} & \sigma_{\lambda\nu} & \sigma_{\nu\nu} & \sigma_\nu^2 \end{pmatrix}, \\
\sigma_{\lambda\lambda} &= \rho_\lambda \sigma_\lambda^2, \\
\sigma_{\nu\nu} &= \rho_\nu \sigma_\nu^2, \\
\sigma_{\lambda\nu} &= \rho_{\lambda\nu} \sigma_\lambda \sigma_\nu,
\end{aligned}
$$

where ρ_λ and ρ_ν are within-measure correlations representing the test–retest reliability of either measure, and $\rho_{\lambda\nu}$ is the between-measure correlation representing the convergent validity. Since not all values of ρ_λ, ρ_ν and $\rho_{\lambda\nu}$ in the range $[-1, 1]$ result in a valid covariance matrix $\boldsymbol{\Sigma}$, instead of parameterizing $\boldsymbol{\Sigma}$ directly, we parameterize the Cholesky factor of the

correlation matrix[2]:

$$
\begin{aligned}
\boldsymbol{\Sigma} &= \operatorname{diag}(\boldsymbol{\sigma})\boldsymbol{P}\operatorname{diag}(\boldsymbol{\sigma})\,, \\
\boldsymbol{\sigma} &= (\sigma_\lambda, \sigma_\lambda, \sigma_\nu, \sigma_\nu)^\top\,, \\
\boldsymbol{P} &= \boldsymbol{L}\boldsymbol{L}^\top\,, \\
\boldsymbol{L} &= \begin{pmatrix} 1 & 0 & 0 & 0 \\ a_1 & A_1 & 0 & 0 \\ a_2 & A_2 a_3 & A_2 A_3 & 0 \\ a_2 & A_2 a_3 & A_2 A_3 a_4 & A_2 A_3 A_4 \end{pmatrix}, \\
a_j &\in [-1, 1]\,, \\
A_j &= \sqrt{1 - a_j^2}\,, \\
a_3 &= \frac{(1 - a_1) a_2}{A_1 A_2}\,,
\end{aligned}
$$

where $a_{\{1,2,4\}}$ are the true parameters for the correlation matrix $\boldsymbol{P}$. Such parameterization guarantees that $\boldsymbol{\Sigma}$ is a valid covariance matrix and that its constraints are satisfied.

We set the priors to be

$$
\begin{aligned}
\mu_\lambda &\sim N(0, 2)\,, \\
\mu_\nu &\sim N(20, 40)\,, \\
\sigma_\lambda &\sim \operatorname{Lognormal}\big(\ln(1), 1\big)\,, \\
\sigma_\nu &\sim \operatorname{Lognormal}\big(\ln(20), 1\big)\,, \\
\boldsymbol{P} &\sim \operatorname{LKJ}(1)\,.
\end{aligned}
$$

Data points that lie on the boundaries are treated as censored data. We fit the model using RStan with the default sampling parameters.

The ellipses in Fig 4C correspond to a bivariate normal distribution with mean vector $(\mu_\lambda, \mu_\nu)^\top$ and covariance matrix

$$
\begin{pmatrix} \sigma_\lambda^2 & \sigma_{\lambda\nu} \\ \sigma_{\lambda\nu} & \sigma_\nu^2 \end{pmatrix}
$$

where the parameters are set to their posterior medians.

[2]The correlation matrix is written $\boldsymbol{P}$, the Greek capital letter of ρ. Since it looks identical to the Latin letter P, it can be pronounced as either rho or P.

1.3 λ vs. social distance

Let i index the participants; n be the number of distinct social distance rankings minus 1 (9 for the Lambda Slider and 3 for the SVO Slider Measure); $0 \le t \le n$ index the targets sorted by their social distance rankings ($t = 0$ corresponds to the target with the smallest social distance); $r_t \in [0, n]$ be the linear predictor term derived from the monotonic predictor t and the simplex parameters ζ_i, $1 \le i \le n$ [3]; and y_{it} be the dependent variable (λ or SVO°) for participant i and target t. The model is

$$y_{it} \sim N\big(b_1 + b_{2i} + (b_3 + b_{4i})r_t, \sigma_0\big),$$
$$\begin{pmatrix} b_{2i} \\ b_{4i} \end{pmatrix} \sim N\left(\mathbf{0}, \begin{pmatrix} \sigma_2^2 & \rho\sigma_2\sigma_4 \\ \rho\sigma_2\sigma_4 & \sigma_4^2 \end{pmatrix}\right).$$

Values of y_{it} that lie on the boundaries are treated as censored data. The priors for the Lambda Slider data are

$$\begin{aligned}
b_1 &\sim N(1, 2), \\
b_3 &\sim N(0, 0.5), \\
\sigma_0 &\sim \text{Lognormal}(0, 1), \\
\sigma_2 &\sim \text{Lognormal}(0, 1), \\
\sigma_4 &\sim \text{Lognormal}(-1, 1), \\
\rho &\sim \text{Uniform}(-1, 1), \\
\boldsymbol{\zeta} &\sim \text{Dirichlet}(\mathbf{1}).
\end{aligned}$$

The priors for the SVO Slider Measure data are

$$\begin{aligned}
b_1 &\sim N(40, 40), \\
b_3 &\sim N(0, 30), \\
\sigma_0 &\sim \text{Lognormal}(3, 1), \\
\sigma_2 &\sim \text{Lognormal}(3, 1), \\
\sigma_4 &\sim \text{Lognormal}(2, 1), \\
\rho &\sim \text{Uniform}(-1, 1), \\
\boldsymbol{\zeta} &\sim \text{Dirichlet}(\mathbf{1}).
\end{aligned}$$

We fit the model using brms [4] with the default sampling parameters.

2 Experiment 2

2.1 Adjudicating between the two hypotheses

For each participant–target combination i, the two raw slider positions on each of the three sliders ("b" for base, "p" for positive-shift, "n" for negative-shift) form a data vector $\boldsymbol{x}_i$. The data vectors are assumed to be sampled i.i.d. from a 6-variate normal distribution with certain constraints:

$$
\begin{aligned}
\boldsymbol{x}_i &\sim N(\boldsymbol{\mu}, \boldsymbol{\Sigma}), \\
\boldsymbol{x}_i &= (\chi_{i\mathrm{b}1}, \chi_{i\mathrm{b}2}, \chi_{i\mathrm{p}1}, \chi_{i\mathrm{p}2}, \chi_{i\mathrm{n}1}, \chi_{i\mathrm{n}2})^\top, \\
\boldsymbol{\mu} &= (\mu, \mu, \mu - \mu^\Delta, \mu - \mu^\Delta, \mu + \mu^\Delta, \mu + \mu^\Delta)^\top, \\
\boldsymbol{\Sigma} &= \sigma^2 \begin{pmatrix}
1 & \rho_\mathrm{b} & \rho_\mathrm{bp} & \rho_\mathrm{bp} & \rho_\mathrm{bn} & \rho_\mathrm{bn} \\
\rho_\mathrm{b} & 1 & \rho_\mathrm{bp} & \rho_\mathrm{bp} & \rho_\mathrm{bn} & \rho_\mathrm{bn} \\
\rho_\mathrm{bp} & \rho_\mathrm{bp} & 1 & \rho_\mathrm{p} & \rho_\mathrm{pn} & \rho_\mathrm{pn} \\
\rho_\mathrm{bp} & \rho_\mathrm{bp} & \rho_\mathrm{p} & 1 & \rho_\mathrm{pn} & \rho_\mathrm{pn} \\
\rho_\mathrm{bn} & \rho_\mathrm{bn} & \rho_\mathrm{pn} & \rho_\mathrm{pn} & 1 & \rho_\mathrm{n} \\
\rho_\mathrm{bn} & \rho_\mathrm{bn} & \rho_\mathrm{pn} & \rho_\mathrm{pn} & \rho_\mathrm{n} & 1
\end{pmatrix},
\end{aligned}
$$

where $\rho_{\{\mathrm{b,p,n}\}}$ are within-slider correlations, and $\rho_{\{\mathrm{bp,bn,pn}\}}$ are between-slider correlations. According to Eqs (9) and (10), we have $\mu^\Delta = 0.1875$ for H_λ and $\mu^\Delta = 0$ for H_χ.

Like above, not all values of $\rho_{\{\mathrm{b,p,n,bp,bn,pn}\}}$ in the range $[-1, 1]$ result in a valid covariance

matrix $\boldsymbol{\Sigma}$, so again we parameterize the Cholesky factor of the correlation matrix:

$$\begin{aligned}
\boldsymbol{\Sigma} &= \sigma^2 \boldsymbol{P} = \sigma^2 \boldsymbol{L}\boldsymbol{L}^\top , \\
\boldsymbol{L} &= \begin{pmatrix}
1 & 0 & 0 & 0 & 0 & 0 \\
a_1 & A_1 & 0 & 0 & 0 & 0 \\
a_2 & A_2 a_4 & A_2 A_4 & 0 & 0 & 0 \\
a_2 & A_2 a_4 & A_2 A_4 a_6 & A_2 A_4 A_6 & 0 & 0 \\
a_3 & A_3 a_5 & A_2 A_5 a_7 & A_2 A_5 A_7 a_8 & A_2 A_5 A_7 A_8 & 0 \\
a_3 & A_3 a_5 & A_2 A_5 a_7 & A_2 A_5 A_7 a_8 & A_2 A_5 A_7 A_8 a_9 & A_2 A_5 A_7 A_8 A_9
\end{pmatrix} , \\
a_j &\in [-1, 1] , \\
A_j &= \sqrt{1 - a_j^2} , \\
a_4 &= \frac{(1 - a_1) a_2}{A_1 A_2} , \\
a_5 &= \frac{(1 - a_1) a_3}{A_1 A_3} , \\
a_8 &= \frac{(1 - a_6) a_7}{A_6 A_7} ,
\end{aligned} \tag{17}$$

where $a_{\{1,2,3,6,7,9\}}$ are the true parameters for the correlation matrix $\boldsymbol{P}$. Such parameterization guarantees that $\boldsymbol{\Sigma}$ is a valid covariance matrix and that its constraints are satisfied.

We set the priors to be

$$\begin{aligned}
\mu &\sim N(0.5, 0.5) , \\
\sigma &\sim \text{Lognormal}\big(\ln(0.25), 1\big) , \\
\boldsymbol{P} &\sim \text{LKJ}(1) .
\end{aligned}$$

Data points that lie on the boundaries are treated as censored data. We fit both models (H_λ and H_χ) using RStan with the default sampling parameters, except that the number of total iterations is set to 5000, half of which are warm-up samples. We estimate the marginal likelihoods of the two models using bridge sampling [5].

In Fig 7, using the base-pos comparison as an example (panel A), the ellipses correspond to a bivariate normal distribution with mean vectors $(\mu, \mu - \mu^\Delta)^\top$ and covariance matrix

$$\sigma^2 \begin{pmatrix} 1 & \rho_{\text{bp}} \\ \rho_{\text{bp}} & 1 \end{pmatrix}$$

where the parameters are set to their posterior medians.

2.2 λ vs. social distance

The model is similar to Experiment 1, but with an extra predictor representing the slider, which we assume only changes the intercept but not the slope on r_t. Let s_p and s_n be dummy variables corresponding to the positive- and negative-shift sliders. The model becomes

$$y_{it} \sim N\big(b_1 + b_{2i} + b_5 s_\mathrm{p} + b_6 s_\mathrm{n} + (b_3 + b_{4i}) r_t, \sigma_0\big),$$

and the priors are the same as the ones for the Lambda Slider data in Experiment 1, with the extra terms

$$b_5 \sim N(0,1),$$
$$b_6 \sim N(0,1).$$

Note that the dependent variable is slider position x, whose range depends on the slider, not the *raw* slider position χ, whose range is always $[0,1]$.

3 Experiment 3

3.1 Robustness

For each participant–target combination i, the two slider positions on each of the three sliders ("b" for balanced, "s" for self-more, "t" for target-more) form a data vector $\boldsymbol{x}_i$. The data vectors are assumed to be sampled i.i.d. from a 6-variate normal distribution with

certain constraints:

$$
\begin{aligned}
\boldsymbol{x}_i &\sim N(\boldsymbol{\mu}, \boldsymbol{\Sigma}), \\
\boldsymbol{x}_i &= (x_{i\mathrm{b}1}, x_{i\mathrm{b}2}, x_{i\mathrm{s}1}, x_{i\mathrm{s}2}, x_{i\mathrm{t}1}, x_{i\mathrm{t}2})^\top, \\
\boldsymbol{\mu} &= (\mu_\mathrm{b}, \mu_\mathrm{b}, \mu_\mathrm{s}, \mu_\mathrm{s}, \mu_\mathrm{t}, \mu_\mathrm{t})^\top, \\
\boldsymbol{\Sigma} &= \begin{pmatrix}
\sigma_\mathrm{b}^2 & \sigma_\mathrm{bb} & \sigma_\mathrm{bs} & \sigma_\mathrm{bs} & \sigma_\mathrm{bt} & \sigma_\mathrm{bt} \\
\sigma_\mathrm{bb} & \sigma_\mathrm{b}^2 & \sigma_\mathrm{bs} & \sigma_\mathrm{bs} & \sigma_\mathrm{bt} & \sigma_\mathrm{bt} \\
\sigma_\mathrm{bs} & \sigma_\mathrm{bs} & \sigma_\mathrm{s}^2 & \sigma_\mathrm{ss} & \sigma_\mathrm{st} & \sigma_\mathrm{st} \\
\sigma_\mathrm{bs} & \sigma_\mathrm{bs} & \sigma_\mathrm{ss} & \sigma_\mathrm{s}^2 & \sigma_\mathrm{st} & \sigma_\mathrm{st} \\
\sigma_\mathrm{bt} & \sigma_\mathrm{bt} & \sigma_\mathrm{st} & \sigma_\mathrm{st} & \sigma_\mathrm{t}^2 & \sigma_\mathrm{tt} \\
\sigma_\mathrm{bt} & \sigma_\mathrm{bt} & \sigma_\mathrm{st} & \sigma_\mathrm{st} & \sigma_\mathrm{tt} & \sigma_\mathrm{t}^2
\end{pmatrix}, \\
\sigma_{ll} &= \rho_l \sigma_l^2, \quad l \in \{\mathrm{b}, \mathrm{s}, \mathrm{t}\}, \\
\sigma_{l_1 l_2} &= \rho_{l_1 l_2} \sigma_{l_1} \sigma_{l_2}, \quad l_1 l_2 \in \{\mathrm{bs}, \mathrm{bt}, \mathrm{st}\},
\end{aligned}
$$

where ρ_l are within-slider correlations, and $\rho_{l_1 l_2}$ are between-slider correlations. The parameterization of $\boldsymbol{\Sigma}$ (in fact, the correlation matrix $\boldsymbol{P}$) is the same as in Experiment 2, which was not described in the preregistration because we were not aware of the issue that not all values of ρ result in a valid covariance matrix.

For the alternative model where $\rho_\mathrm{b} = \rho_\mathrm{s} = \rho_\mathrm{t}$, the Cholesky factor of $\boldsymbol{P}$ has the same format as Eq (17), but with two additional constraints among the variables:

$$
\begin{aligned}
a_6 &= \frac{a_1 - a_2^2 - A_2^2 a_4^2}{A_2^2 A_4^2}, \\
a_9 &= \frac{a_1 - a_3^2 - A_3^2 a_5^2 - A_3^2 A_5^2 a_7^2 - A_3^2 A_5^2 A_7^2 a_8^2}{A_3^2 A_5^2 A_7^2 A_8^2},
\end{aligned}
$$

so $a_{\{1,2,3,7\}}$ are the true parameters for $\boldsymbol{P}$.

We set the priors to be

$$
\begin{aligned}
\mu_l &\sim N(0, 2), \\
\sigma_l &\sim \mathrm{Lognormal}(0, 1), \\
\boldsymbol{P} &\sim \mathrm{LKJ}(1).
\end{aligned}
$$

Data points that lie on the boundaries are treated as censored data. We fit the full model using RStan with the default sampling parameters, except that the number of total iterations is set to 3000, half of which are warm-up samples. For estimating the Bayes factor

between the two models, we randomly sample 10% of the data (bridge sampling would not reliably converge for more data, likely because there are too many parameters in the model corresponding to the censored data points) and fit the two models with the default sampling parameters, except that the number of total iterations is set to 5000, half of which are warm-up samples. We repeat this process 10 times, producing 10 Bayes factors, and report their median in the main text.

For the relationship between λ and social distance, the model is similar to Experiment 2, but with a different set of sliders and two extra predictors—sex and the interaction between sex and social distance. Let s_s and s_t be dummy variables corresponding to the self-more and target-more sliders. Let s_m be dummy variable corresponding to being male (instead of female). The model becomes

$$y_{it} \sim N\big(b_1 + b_{2i} + b_5 s_\mathrm{s} + b_6 s_\mathrm{t} + b_7 s_\mathrm{m} + b_8 s_\mathrm{m} r_t + (b_3 + b_{4i}) r_t, \sigma_0\big),$$

and the priors are the same in Experiment 2, with the extra terms

$$\begin{aligned} b_7 &\sim N(0, 0.5), \\ b_8 &\sim N(0, 0.2). \end{aligned}$$

The number of total sampling iterations is 5000, half of which are warm-up samples.

Call this full model M_1. There are two alternative models, the first one without the b_8 term (call it M_2), and the second one without both b_7 and b_8 (call it M_3). M_3 is essentially the same model as in Experiment 2. The mean slope b_3 reported in the main text is from M_3 fit to the full dataset. To examine the effect of sex, we fit the three models to a dataset where the only participant whose sex was "prefer not to say" is excluded. There is very weak evidence for the *nonexistence* of an interaction between sex and social distance ($b_8 = 0.05$ $(-0.16, 0.26)$, $\mathrm{BF}_{M_1/M_2} = 0.55$) and no evidence for the existence or nonexistence of an effect of sex ($b_7 = 0.16$ $(-0.16, 0.47)$, $\mathrm{BF}_{M_2/M_3} = 0.91$).

3.2 External validity

For each participant i, the two measurements on a particular Lambda Slider toward the Maui wildfires victim $x_{i\{1,2\}} \in [-2, 2]$ and the participant's donation to the Maui Strong Fund $d_i \in [0, 2]$ form a data vector $\boldsymbol{x}_i$. The data vectors are assumed to be sampled i.i.d.

from a 3-variate normal distribution with certain constraints:

$$
\begin{aligned}
\boldsymbol{x}_i &\sim N(\boldsymbol{\mu}, \boldsymbol{\Sigma}), \\
\boldsymbol{x}_i &= (x_{i1}, x_{i2}, d_i)^\top, \\
\boldsymbol{\mu} &= (\mu_\lambda, \mu_\lambda, \mu_d)^\top, \\
\boldsymbol{\Sigma} &= \begin{pmatrix} \sigma_\lambda^2 & \rho_\lambda \sigma_\lambda^2 & \rho_{\lambda d}\sigma_\lambda\sigma_d \\ \rho_\lambda \sigma_\lambda^2 & \sigma_\lambda^2 & \rho_{\lambda d}\sigma_\lambda\sigma_d \\ \rho_{\lambda d}\sigma_\lambda\sigma_d & \rho_{\lambda d}\sigma_\lambda\sigma_d & \sigma_d^2 \end{pmatrix}.
\end{aligned}
$$

The Cholesky factor of the correlation matrix is parameterized as

$$
\begin{aligned}
\boldsymbol{L} &= \begin{pmatrix} 1 & 0 & 0 \\ a_1 & A_1 & 0 \\ a_2 & A_2 a_3 & A_2 A_3 \end{pmatrix}, \\
a_j &\in [-1, 1], \\
A_j &= \sqrt{1 - a_j^2}, \\
a_3 &= \frac{(1 - a_1) a_2}{A_1 A_2},
\end{aligned}
$$

where the true parameters are $a_{\{1,2\}}$. In the null model where $\rho_{\lambda d} = 0$, there is an additional constraint $a_2 = 0$, and the only true parameter is a_1.

We set the priors to be

$$
\begin{aligned}
\mu_\lambda &\sim N(0, 2), \\
\mu_d &\sim N(1, 1), \\
\sigma_\lambda, \sigma_d &\sim \text{Lognormal}(0, 1), \\
\boldsymbol{P} &\sim \text{LKJ}(1).
\end{aligned}
$$

Data points that lie on the boundaries are treated as censored data. We fit both the full model and the null model using RStan with the default sampling parameters, except that the number of total iterations is set to 5000, half of which are warm-up samples. We estimate the marginal likelihoods of the two models using bridge sampling.

3.3 Inequity aversion

We assume that participants' choices on the sliders are noisy maximization of the utility function:

$$p(x) \propto \exp\big(\beta u(x)\big), \tag{18}$$

where $\beta > 0$ is a global softmax parameter (we do not have enough data to fit a β for each participant). Substituting Eq (12) into Eq (18), we see that $p(x)$ has the same form as the probability density function of a normal distribution on each segment of the slider where $w_\mathrm{s} - w_\mathrm{t}$ has the same sign. In other words, x is distributed according to a truncated normal distribution on each of these segments, with the constraint that $p(x)$ is continuous at the boundaries between segments.

Let $\phi_{\mu,\sigma}$ and $\Phi_{\mu,\sigma}$ be the density and cumulative functions of a normal distribution with mean μ and standard deviation σ. Let

$$\begin{aligned}
\mu_1 &= \frac{\lambda+\kappa}{1-\kappa}, \\
\mu_2 &= \frac{\lambda-\kappa}{1+\kappa}, \\
\sigma_1 &= \frac{1}{2\beta a(1-\kappa)}, \\
\sigma_2 &= \frac{1}{2\beta a(1+\kappa)},
\end{aligned}$$

where $a = 7$ is the scale of the slider. The likelihood functions for the self-more and target-more sliders are

$$\begin{aligned}
p_\mathrm{s}(x \mid \lambda,\kappa,\beta) &= \frac{\phi_{\mu_1,\sigma_1}(x)}{\Phi_{\mu_1,\sigma_1}(2) - \Phi_{\mu_1,\sigma_1}(-2)}, \\
p_\mathrm{t}(x \mid \lambda,\kappa,\beta) &= \frac{\phi_{\mu_2,\sigma_2}(x)}{\Phi_{\mu_2,\sigma_2}(2) - \Phi_{\mu_2,\sigma_2}(-2)}.
\end{aligned}$$

The likelihood function for the balanced slider is slightly more complex because there are two segments where $w_\mathrm{s} - w_\mathrm{t}$ has different signs:

$$p_\mathrm{b}(x \mid \lambda,\kappa,\beta) = \begin{cases} \dfrac{\phi_{\mu_1,\sigma_1}(x)}{A_1 + kA_2} & x \in [-2, \hat{x}] \\ \dfrac{\phi_{\mu_2,\sigma_2}(x)}{\frac{1}{k}A_1 + A_2} & x \in (\hat{x}, 2] \end{cases},$$

where

$$\begin{aligned}
\hat{x} &= \sqrt{3}-1\,,\\
A_1 &= \Phi_{\mu_1,\sigma_1}(\hat{x}) - \Phi_{\mu_1,\sigma_1}(-2)\,,\\
A_2 &= \Phi_{\mu_2,\sigma_2}(2) - \Phi_{\mu_2,\sigma_2}(\hat{x})\,,\\
k &= \frac{\phi_{\mu_1,\sigma_1}(\hat{x})}{\phi_{\mu_2,\sigma_2}(\hat{x})}\,.
\end{aligned}$$

Unless $\lambda = -1$, both $\mu_1 \to \infty$ and $\sigma_1 \to \infty$ when $\kappa \to 1$, which makes the model unstable, so we restrict the range of κ to $[0, 0.95]$.

Let i index the participants; κ_i be a participant's κ parameter; $1 \le t \le 6$ index the targets; λ_{it} be the λ parameter for a participant–target combination; and y_{itl} be the response for participant i, target t, and slider l. The model is

$$\begin{aligned}
\lambda_{it} &\sim N(\mu_t, 0.5)\\
y_{itl} &\sim p_l(\cdot \mid \lambda_{it}, \kappa_i, \beta)\,,
\end{aligned}$$

where μ_t is the mean λ for a target across participants. We put a slightly strong prior on λ_{it} (but not unreasonable given prior data) because for extreme values of λ, the posterior distribution would be strongly degenerate and the sampling algorithm would have difficulty exploring the distribution efficiently. The other priors are

$$\begin{aligned}
\mu_t &\sim N(0,1)\,,\\
\beta &\sim \text{Lognormal}(-1, 0.5)\,,\\
\kappa_i &\sim \text{Uniform}(0, 0.95)\,.
\end{aligned}$$

The relatively strong prior on β is also set to prevent degeneracy. We fit the model using RStan with the default sampling parameters.

The correlation between κ_i and $|d_i - 1|$ (where d_i is the donation amount) is calculated

by fitting a bivariate normal distribution to the data, without treating any data as censored:

$$\begin{aligned}
\boldsymbol{x}_i &\sim N(\boldsymbol{\mu}, \boldsymbol{\Sigma}), \\
\boldsymbol{x}_i &= \left(\kappa_i, |d_i - 1|\right)^\top, \\
\boldsymbol{\mu} &= (\mu_1, \mu_2)^\top, \\
\boldsymbol{\Sigma} &= \begin{pmatrix} \sigma_1^2 & \rho\sigma_1\sigma_2 \\ \rho\sigma_1\sigma_2 & \sigma_2^2 \end{pmatrix},
\end{aligned}$$

and the priors are

$$\begin{aligned}
\mu_1, \mu_2 &\sim N(0.5, 0.5), \\
\sigma_1, \sigma_2 &\sim \text{Lognormal}\left(\ln(0.5), 1\right), \\
\rho &\sim \text{Uniform}(-1, 1).
\end{aligned}$$

We fit the model using RStan with the default sampling parameters.

References

1. Stan Development Team. Stan Modeling Language Users Guide and Reference Manual; 2023. Available from: https://mc-stan.org/.
2. Stan Development Team. RStan: the R interface to Stan; 2024. Available from: https://mc-stan.org/.
3. Bürkner PC, Charpentier E. Modelling monotonic effects of ordinal predictors in Bayesian regression models. British Journal of Mathematical and Statistical Psychology. 2020;73(3):420–451.
4. Bürkner PC. brms: An R package for Bayesian multilevel models using Stan. Journal of Statistical Software. 2017;80:1–28.
5. Gronau QF, Singmann H, Wagenmakers EJ. bridgesampling: An R package for estimating normalizing constants. Journal of Statistical Software. 2020;92:1–29.
