# Supplementary material for: An accurate and efficient measure of welfare tradeoff ratios
Source: PLoS One. 2025 May 27;20(5):e0322410. doi: 10.1371/journal.pone.0322410 (PMC12112420; doi:10.1371/journal.pone.0322410)
Supplement: Attachment — Submitted filename: response-plos.pdf [file pone.0322410.s005.pdf]

Dear Editor Ridinger,

Thank you for offering us the opportunity to resubmit a revised version of our manuscript, and thanks to you and the two reviewers for insightful comments and suggestions. Below is a point-by-point response to all the questions and issues raised by you and the reviewers, where the sentences in red describe the changes we have made to the manuscript. All references to the manuscript refer to the revised version.

Editor

> Thank you for submitting your manuscript to PLOS ONE. After careful consideration, we feel that it has merit but does not fully meet PLOS ONE's publication criteria as it currently stands. Therefore, we invite you to submit a revised version of the manuscript that addresses the points raised during the review process.
>
> I have received referee reports from two experts in this area of research and they have provided excellent feedback. Reviewer 1 suggests a major revision. Reviewer 2 suggests a minor revision. I would like to echo Reviewer 2 in that I think this paper has great potential to make a nice contribution to the literature but would benefit from addressing the concerns in the referee reports. I encourage the authors to address each of the reviewers suggestions and comments. Additionally, I have some suggestions that I think may help as well. Below I highlight a few main areas I encourage the authors to address in the revision:

Thank you for your kind assessment of our work. We hope we have adequately addressed the concerns from you and the reviewers.

> Sample size: Reviewer 1 discussed a concern that both experiment 1 and 2 have a very small sample sizes. The authors should address those comments. Additionally, more information is needed about how the sample size for the first experiments were determined. While the authors are correct that Bayesian analysis can handle small samples, this means that the prior will have a stronger influence on the results. It would be helpful for the authors to discuss their prior and how it was determined. Was the prior chosen as weakly informative or based on prior SVO data? More discussion is needed. It may be helpful to run a post-hoc power analysis to see the degree in which the first two experiments may be underpowered. Additionally, were the results from experiments 1 and 2 used for the power analysis for the preregistration for experiment 3? More details would be helpful.

We admit that the sample sizes of the first two experiments were not large in the context of contemporary social psychology studies nor were they determined based on formal power

analyses (we mention in footnote 7 that the samples sizes were determined “heuristically”). The probability of making a type II error using NHST might a priori be large. However, after the data were analyzed, since all the alternative hypotheses were strongly preferred to the null hypotheses (e.g., a non-zero correlation versus a zero correlation), type II errors and underpowering are no longer a concern. From a Bayesian point of view, the credible intervals of the estimated parameters are tight enough for our purposes, so it did not seem beneficial to collect more data, particularly in light of the subsequent experiments; Experiment 3 had a larger, preregistered sample size and confirmed the test-retest reliability and strong correlation with social distance.

We agree that with smaller sample sizes, the priors will have a stronger influence on the results. Therefore, as documented in S4 Appendix, we chose uninformative or weakly informative priors (based on the null hypotheses) for the estimated parameters. For instance, for the test–retest correlation (ρ), we used a uniform distribution between -1 and 1. For the slope between λ and social distance ranking (b_3), we used a normal distribution with mean 0 and standard deviation 0.5, which is very weakly informative given the reasonable range of slopes we can expect (the final estimate was $b_3 = -0.25$). These priors contain either no bias or a weak bias toward the null hypotheses, which should not be a concern when the alternative hypotheses are strongly preferred to the null hypotheses. We mentioned this in the second paragraph of Experiment 1/Results/Test–retest reliability.

Results from Experiments 1 and 2 were not used for the power analysis for the preregistration for Experiment 3 because there was no correlation in Experiment 1 or 2 that was similar to the correlation between Lambda Slider measurements and the donation amounts in Experiment 3. As mentioned in the preregistration, we aimed to detect medium effect sizes of $r = 0.3$.

> Experimental Instructions: I tried out the lamda task experiment using the link provided by the authors in the text. One issue I saw is that in the non-zero sum distribution questions, the only way a subject will know the full range of choices is if they move the slider in both directions. It is not clear in the directions that subjects should try to move the slider in both directions for the full range to check their choices. In the software is there any recording whether subjects moved the slider the full range to see all the possible distribution options? If so, then this data may be worth checking to see if this introduced any bias in the elicitation method. Additionally, it may be worth discussing this as a possible drawback as the elicitation method may introduce some error in elicitation if some subjects do not move the slider to check the full range of choices.

Thanks for pointing this out. Unfortunately, we did not record exactly how participants

moved the slider and whether they moved the slider the full range, which could be useful data to analyze. However, even if participants did not move the slider the full range, this may not have been a source of bias in the measurements, for three reasons. First, as long as participants are maximizing a utility function in the form of Eq (11) in the manuscript (the definition of WTR plus the inequity-aversion term), they do not have to check the full range of choices to find the global maximum of their utility function. They only need to follow the gradient of the utility function, which has only one peak for any λ and $\kappa \in [0, 1)$. Second, the starting position of the slider was randomized across trials, so regardless of participants' own behavior they necessarily observed relative payoffs in different portions of the slider as the study progressed. Third, it is plausible that after being familiarized with the Lambda Slider in the first few trials, participants establish a sense of the mapping between slider positions and WTRs and can restrict their search for the best choice to a portion of the range, especially when the payoff structure of the Lambda Slider is fixed, like in Experiment 1. We did, however, add a paragraph in the Discussion to suggest analyzing the fine-grained movement data in future studies to test these hypotheses and to assess whether instructions to explore the slider could improve reliability.

> Comparison to SVO: Both reviewers highlight that the paper would benefit from more discussion about how the lamda slider compares and contrasts to SVO.

There are two comparisons that can be made: WTR vs. SVO as psychological constructs, and the Lambda Slider vs. the SVO Slider Measure as measures.

We believe that fundamentally WTR and SVO are the same thing. Their difference is mostly historical—SVO was traditionally treated as categorical and describes a person's stable disposition toward a unidentified other, while WTR is usually treated as continuous and specific to each decision influenced by a variety of factors. Recent work has shown a convergence between these two concepts (e.g., [1, 2]). We added a footnote in the "binary allocation task" section to clarify this.

As suggested by Reviewer 1, we now also bring up SVO earlier in the Introduction and note that it has the same accuracy–efficiency tradeoff issue as existing WTR measures.

> Data Analysis: Reviewer 2 has some great suggestions about providing more data analysis and suggests adding some descriptive statistics for each experiment. There were multiple measures in the experiment and provided descriptive statistics for each measure would help.

We address this at the corresponding points below.

Reviewer 1

> The authors introduce the Lambda Slider, a novel measure of social preferences, based on a welfare tradeoff ratio (WTR). The major benefit of this measure is that it uses a single continuous slider to give a more precise measure of the concern for others, which would require participants to make multiple binary allocations in previous methods such as the SVO.
>
> Experiment 1 tests the reliability of the Lambda Slider and SVO by eliciting two measurements for each participant. The authors find that correlations between the two measurements are high for both the Lambda Slider and SVO. The two measures are also positively correlated (convergent validity). Experiment 2 provides a robustness check and shows that participants are not simply moving the position of the Lambda Slider without any regard for the corresponding allocation values. Experiment 3 tests external validity by asking participants to make a donation. The authors show that the measure is positively correlated with donations. Experiment 3 also cautions that the measure may be biased by inequality aversion.
>
> The paper presents a measure that is useful to researchers studying social preferences with nice benefits over existing methods. I appreciate the robustness check conducted in Experiment 2 as well as the test of external validity using an incentivized charitable donation in Experiment 3.

Thank you for your accurate summary and favorable evaluation of our work.

> However, my main concern is with the sample sizes of Experiment 1 and 2. In Exp 1 only 40 participants were recruited with 30 remaining after excluding those who failed attention checks. While in Exp 2 only 20 were recruited with only 16 passing the checks. Given that the reliability of the Lambda Slider is a major selling point and a central part of the paper, I am surprised that so few observations were collected. While for Experiment 3 (which I understand was the only preregistered experiment), 90 observations were collected.

We agree that the sample sizes of Experiments 1 and 2 are small compared to other social psychological studies, but we believe that they are adequate because of the multiple measurements per participant and the tight credible intervals in the results. These experiments were also intended as initial tests during measure development, and thus were modest in scale. The reliability of the Lambda Slider was verified in Experiment 3 with a larger, preregistered sample size. See also our response to the editor's related comment above.

> When reading the Abstract and Introduction, the contribution of this particular paper was not fully clear to me. This may be personal preference but I found the Introduction to be quite long and took quite a while before any discussion of what is done in this paper and how it differs from

> previous work. I feel that a discussion of the SVO should come much earlier in the Introduction (and should also be mentioned in the Abstract) given that it is what comes to mind for most people for measuring social preferences. Alongside this, I think the authors should emphasise more what sets the lambda slider apart from the SVO and especially the advantages over the SVO.

We agree that the Introduction is long, and that readers would benefit from a brief overview of the contributions of the paper near the beginning, so as to know what to expect. We have revised the beginning of the Introduction to summarize the paper's contribution and to provide a brief overview of the structure of the Introduction and experiments. It also now brings up SVO and notes that the continuous SVO Slider Measure has similar accuracy–efficiency tradeoff issues as existing WTR measures. We also added a brief mention of SVO and its similar tradeoff issue to the abstract. We appreciate your suggestion and hope these edits will help readers more easily grasp the value of our new measure.

> Related to the previous point, when the authors discuss the strengths of the lambda slider it would be helpful to define the criteria discussed throughout the paper in the Introduction, e.g., terms such as reliability, efficiency, accuracy, and sensitivity. This would help to convince the reader early on of the advantages of this method over previous methods.

Thanks for the suggestion. We had clarified the relationship between accuracy, unbiasedness, precision, and reliability in footnote 2, and had provided a definition of sensitivity in the third paragraph of that section. We added a definition of efficiency to footnote 2.

Reviewer 2

> The manuscript introduces a new measure of welfare-tradeoff ratios called the lambda slider. In three experiments, the authors show that the measure has high internal, convergent and external validity, and high reliability. They also find that inequity aversion can bias welfare-tradeoff ratios measurements, but that the new measure can be used to control for inequity aversion.
>
> I thought this was an outstanding paper. In addition to having practical implications for measurement, it is also an elegant and insightful piece of theoretical work. The section on inequity aversion in particular provides very valuable new insights. The logic of the new measure is clearly explained, and the paper is in general very well written. Also the Bayesian analysis and open science practices are exemplary.

> Most of the comments below are motivated by my own curiosity, and I don't think any of them are crucial to the main conclusions of the paper.

Thank you for your compliments. Like you said, we hope our work can provide a useful tool as well as theoretical insights for future studies for the social psychology community.

> It might be useful to the reader if the relationship between SVO and lambda was explicated more precisely. The manuscript says that the SVO measure is "similar" to lambda, but this is ambiguous between "it measures the same thing in a slightly different way" and "it measures a slightly different thing".

As mentioned above in the response to the editor's related comment, we have explicated the relationship between WTR and SVO as theoretical constructs in footnote 4. We also modified the second sentence in the "SVO Slider Measure" section to avoid saying that λ and SVO are "similar", but suggest that they are the same thing.

> I think many readers will be curious about the descriptive statistics. For example, the authors report the average wtrs for each slider in experiment 3, but this could also be reported for the other experiments (as well as the standard deviations). A similar point holds for the descriptive statistics of Dictator Game giving in experiment 3, which I didn't find.

Thank you for the suggestion. We added the means and standard deviations for the main variables in the three experiments.

> One way to measure the reliability of the measure is to assess test-retest reliability, either within or between sliders, as the authors do. But another way is to test to what extent different sliders yield the same average wtr. Suppose for example that two sliders A and B have a .95 between-slider reliability, but mean wtr is .2 when measured by A, and .6 when measured by B. Then there is a sense in which the slider method does not reliably tap into "the true wtr" of a participant. This might of course not be a concern for certain research purposes (for example for experiments only interested in explaining variance in wtr), but could be relevant for others. This issue is one reason why it would be useful to report descriptive statistics for each slider in experiment 2.
>
> On a related note, in experiment 3 the measured wtr is clearly not slider-invariant, as helpfully reported in the text. As the authors convincingly argue, this non-invariance can be explained as a result of inequity aversion. So, if we find that slider-invariance also fails to hold in experiment 2, this might simply mean that inequity aversion is also implicated in that experiment.
>
> To be clear, I think that the issue described above points to a *strength* of the present method. The

> flexibility in the parameterization of the measure allows researchers to explore theoretical issues which are also a concern for other measures but difficult to investigate with these measures.

This is an interesting topic, and, as you mention, we hope we have partially addressed it in Experiment 3. As for Experiment 2, there is clearly violation of slider-invariance in the data—in Fig 7, if slider-invariance holds, the data points would cluster around the H_λ line symmetrically, but even disregarding the points on the boundaries, they are slightly biased toward H_χ, and there is a cluster of points around the equal-payoff points halfway between the two hypotheses. This can also be explained by inequity aversion, and we added a sentence in the first paragraph of the "inequity aversion" section of Experiment 3 to mention this.

> One question raised by Experiment 3 is to what extent we should in general expect inequity aversion to bias our estimates of WTR. The comparison between the three sliders suggests that this bias can be subtantial (the average measured wtrs range from .08 to 1.04). But this comparison doesn't yet tell us whether inequity aversion can bias our measure of relative WTRs. For example one can imagine that when measuring WTR in the "naïve way" we estimate that participant A's wtr is greater than participant B's, but that when controlling for inequity aversion (as the authors do) we find that B's wtr is higher. So it might be instructive to run a "naïve" analysis of experiment 3 (where one just infers wtr instead of wtr and inequity aversion jointly), to see how much the results from that analysis might diverge from the more mature one reported here.

This is a great idea. In Fig 9, the crosses can be interpreted as the naïve measurements we would expect without taking into account inequity aversion. We modified Fig 9 to also show the inferred λ for each participant–target combination, whose vertical distance from the crosses reflect the amount of bias we would expect for different sliders and different "true" WTRs. We also added a discussion about this in the preceding paragraph. In terms of relative WTRs, an example in Fig 9 that matches the situation you describe is panel C, target 4 vs. panel D, target 2 on the balanced slider. The former has a lower "true" WTR (horizontal line) but a higher "naïve" WTR (red cross).

> In experiment 2, some readers might be interested in the between-slider correlations.

We reported the between-slider correlations in Experiment 2.

> One possible direction for future work that could be mentioned is comparison of the lambda slider with the Welfare-Tradeoff Task.

We mentioned this in footnote 5.

> I was impressed by the clarity of the way the slider measure is motivated by imagining adding more and more options to an n-tuple dominance task. Maybe a complementary way of conveying intuition is to remark that each point of the slider corresponds to one particular exchange rate between the participant and the target's welfare. I.e. what makes the measure efficient is that the participant can vary the exchange rate continuously until she finds the one that corresponds to her preferred one. (The authors should feel free to ignore this remark if this makes that section of the paper too long).

Thanks for the suggestion and great phrasing. We added such a remark in the second paragraph of the "Lambda Slider" section.

> when re-deriving the equation for k (between lines 569 and 570) I obtained a slightly different result, k=(x1-x2)/(2+x1+x2). It is very possible the mistake is on my end but it might be worth checking for a possible sign error.

Thanks for catching this, and you are correct. We fixed the error.

> (very minor) I was curious about the pattern of correlations between WTR, inequity aversion, and DG giving. For example if we predict DG giving from WTR and inequity aversion jointly, does one variable come out as a better predictor? But maybe some of these questions are difficult to address rigorously, because experiments are not powered with these analyses in mind and the inequity aversion of a participant is measured from more data points and so potentially more precise.

This is an interesting question. We will not dive into it in the manuscript which is already very long, but here is a sketch of how we can study the question based on the model of Eq (11) in the manuscript. Let λ and κ be the "true" parameters (which can be jointly estimated using multiple Lambda Sliders), x be the measurement on a Lambda Slider with a particular configuration, and d be the DG giving. We can have four models based on Eq (11), with d as the dependent variable:

1. both λ and κ are predictors
2. λ is the only predictor ($\kappa = 0$)
3. κ is the only predictor ($\lambda = 0$)
4. x is the only predictor ($\lambda = x$, $\kappa = 0$)

If model 2 or model 4 is better than model 3 in the sense of having a smaller mean prediction error, we might say that WTR is a better predictor than inequity aversion, but it mostly depends on the distribution of λ and κ in the population. In any case, we would expect that model 1 makes the best predictions.

> (very minor) To help readers keep track of when x (position on a slider) directly maps to lambda, maybe the authors could say that it does so in all experiments unless indicated otherwise.

Thanks for the suggestion. We added a reminder at the end of the "Lambda Slider" section.

References

1. Murphy RO, Ackermann KA. Social value orientation: Theoretical and measurement issues in the study of social preferences. Personality and Social Psychology Review. 2014;18(1):13–41.

2. Ackermann KA, Fleiß J, Murphy RO. Reciprocity as an individual difference. Journal of Conflict Resolution. 2016;60(2):340–367.
