# Supplementary material for: An accurate and efficient measure of welfare tradeoff ratios
Source: PLoS One. 2025 May 27;20(5):e0322410. doi: 10.1371/journal.pone.0322410 (PMC12112420; doi:10.1371/journal.pone.0322410)
Supplement: Attachment — Submitted filename: response-plos-2.pdf [file pone.0322410.s006.pdf]

Dear Editor Ridinger,

Thank you for offering us a second chance to revise our manuscript, and thanks again to Reviewer 2 for continuing to improve our paper with additional helpful comments. Below is a point-by-point response to Reviewer 2's comments, where again the sentences in red describe the changes we have made to the manuscript.

> On p.18 of the revised manuscript (tracked changes version), line 414, I was very confused by the sentence: "Under H_lambda, the mean and standard deviation of X_base are mu=.58 and sd=.27 (Xpos and Xneg have means defined by eq 9 […])".
>
> In my understanding of the setup, X_base is a purely empirical measure (it is the position of the participant's slider for the base slider), so I did not understand why this was contextualized by "under H_lambda". Furthermore, equation 9 describes the predictions of the theory; and saying that we can get the empirical means for Xpos and Xneg in this way seems to already pre-suppose that the theory is right, but checking this is exactly why we are examining the empirical data in the first place. It is instead highly possible that Xpos and Xneg don't have empirical means at exactly the value expected by the theory (even just for simple noise reasons).
>
> I also think the authors should plot these empirical means of lambda for each slider (e.g. as bar graphs) along with the model predictions (for H_lambda and H_X). The Figure with bivariate distributions is great but demands a lot of attention from the reader, the better fit of the H_lambda hypothesis doesn't jump out at the reader immediately.

Thanks for pointing this out. In our last response letter, we failed to make clear that the means and standard deviations we reported were not truly descriptive statistics, but inferred parameters given some model. We opted for the latter because most of our data are bounded by the ranges of the sliders, so the descriptive means and standard deviations do not accurately reflect the central tendency and dispersion of the underlying (censored) data. Of course, this introduces model biases, which are not a major problem in most of our analyses where the model assumptions are reasonable and not the main targets of analysis. But as you pointed out, it is a salient problem here when comparing H_λ and H_χ, and the empirical distributions are particularly useful for this analysis. We added a panel in Fig 7 to plot the empirical distributions under the three sliders and compare them to the model predictions. We also modified the figure caption and main text accordingly, and removed the model-based statistics to reduce confusion.

> On p.24 of the revised manuscript (tracked changes version), the sentence starting with "For the balanced slider […]" (line 561) is potentially confusing because it mentions the descriptive

> results for the balanced slider in the same sentence as the descriptive results for the dictator game, in a way that might implicitly suggest that the dictator game is a “balanced” dictator game and that we should expect the descriptive results for the “self-more” and “other-more” dictator games later (whereas in fact there is only one dictator game). Similarly this sentence reports the average inferred WTR for the balanced slider (which is very useful) but I wasn’t able to find the same information for the other sliders later.

As discussed above, the means and standard deviations we reported were not truly descriptive, and the statistics of both λ and d are relative to a particular model (in this case, the model fit to the balanced slider data). To reduce confusion, we added the same statistics for the self-more and target-more sliders. We can see that the statistics of d are largely invariant with respect to the model. We also corrected an error in reporting μ_d for the balanced slider.

> In response to my comment: “I was curious about the pattern of correlations between WTR, inequity aversion, and DG giving. For example if we predict DG giving from WTR and inequity aversion jointly, does one variable come out as a better predictor? But maybe some of these questions are difficult to address rigorously, because experiments are not powered with these analyses in mind and the inequity aversion of a participant is measured from more data points and so potentially more precise.”, the authors mention a possible approach based on model comparison where one would compare the fit of different regression models including either lambda or kappa as predictors (or both). But I had in mind a simpler and possibly more standard approach where one would simply run a regression of the type DG ∼ lambda + kappa, and compare the magnitude of the standardized coefficients of both predictors.

We did consider this simpler possibility, but we think there are at least two issues that prevent it from being a very meaningful analysis:

1. The theoretical relationship between DG giving ($0 \leq d \leq 2$), λ, and κ is a complex nonlinear one. For instance, when $\lambda < 1$, a higher κ leads to a higher d (switching from $d = 0$ to $d = 1$ at some κ), but when $\lambda > 1$, a higher κ leads to a lower d (switching from $d = 2$ to $d = 1$ at some κ). This issue also makes it less meaningful to compare the pairwise correlations between the three variables.

2. Even assuming that the relationship between d, λ and κ is linear, the presence of multicollinearity makes interpreting the coefficients difficult. For instance, suppose the ground truth is $d = \lambda + \text{noise}$, and λ and κ are highly correlated. Then the regression model $d \sim \lambda + \kappa$ would produce a much smaller coefficient for κ than for λ, but κ by itself is only a slightly worse predictor than λ.

Therefore, we sketched the more complex approach based on model comparisons.
